# Cationic polyacrylamide copolymers (PAMs): environmental half life determination in sludge-treated soil

**DOI:** 10.1186/s12302-018-0143-3

**Published:** 2018-05-18

**Authors:** Dieter Hennecke, Angela Bauer, Monika Herrchen, Erik Wischerhoff, Friedhelm Gores

**Affiliations:** 1 Fraunhofer Institute for Molecular Biology and Applied Ecology, Auf dem Aberg 1, 57392 Schmallenberg, Germany; 2 Fraunhofer Institute for Applied Polymer Research IAP, 14776 Potsdam-Golm, Germany; 3 PSS Polymer Standards Service GmbH, In der Dalheimer Wiese 5, 55120 Mainz, Germany

**Keywords:** Transformation, Sludge-amended soil, Outdoor lysimeter study

## Abstract

**Background:**

Cationic polyacrylamide copolymers (PAMs) are used for sludge dewatering in municipal waste water treatment and might enter the environment by spreading of the sludge on agricultural land. Concern has been expressed since little is known about the degradation of PAMs in soils. To obtain detailed information on the polymer’s fate in the soil compartment, the degradation of ^14^C-radiolabelled PAM in an outdoor lysimeter was studied.

**Results:**

No plant uptake and leaching of radioactivity was observed. There was practically no vertical movement of polymer and no transformation products found at the end of the study. For the top 10 cm soil layer, a mass balance was established throughout the study. About 10% of applied radioactivity was not extractable from soil even with a matrix destructive method, and this was concluded to be bound residue. Characterization of extractable radioactivity by means of GPC-analysis showed a significant decrease of the molecular weight of the PAM with time. The decrease in molecular weight indicates a breakdown of the polymer backbone (the C–C-chain), and is assumed to be primary degradation. The total radioactivity content in the 10 cm top soil layer was quantified every 6 months over a period of 3 years. The results show a significant decrease of the total radioactivity over time and this is defined as ultimate degradation following the definition of OECD and EPA. Based on the data, a half-life time of 2.0 × 10^3^ days and a rate constant of 0.00035/day were calculated. With a *χ*^2^ of 12.0 the results of the calculation are thus valid and reliable. The rate constant indicates a mineralization of 22.5% within a period of 2 years based on the total recovered radioactivity. This half-life time is solely based on mineralization and does not take into account the degradation of the polymer backbone, hydrolysis of the side chains, incorporation into the soil matrix, and thus is a conservative approach.

**Conclusions:**

^14^C-PAM degrades very slowly in soil after land-spreading as a component of sewage sludge. Even in a very conservative evaluation which only considered the loss of radioactivity, a half-life time of 5.4 years was determined.

**Electronic supplementary material:**

The online version of this article (10.1186/s12302-018-0143-3) contains supplementary material, which is available to authorized users.

## Background

Cationic polyacrylamide copolymers (PAMs) are a group of water-soluble polymers with a wide range of applications in industry, food processing, agriculture and waste management. One of the major applications for PAM is sludge dewatering in municipal waste water treatment plants (MWWTPs). Spreading of the sludge on agricultural land is currently one of the most important recycling routes. In Germany, the maximum application rate of sewage sludge on agricultural land is 5 tons dry solid (TDS) per hectare as an average over 3 years [16]. Considering that the dewatered sludge contains around 5 kg/TDS significant amounts of PAM end up on the soil.

As part of a terrestrial risk assessment, the fate of PAMs, i.e., sorption, mobility and abiotic and biotic transformation, needs to be addressed. PAMs are strongly bound to organic matter and clay particles, and are therefore, immobile in soil and very difficult to desorb. The adsorption process occurs rapidly and is mostly irreversible, although the degree of adsorption is influenced by PAM conformation, soil and mineral properties and soil solution characteristics [1]. In general, increasing molecular size and increasing chain extension lead to increased adsorption [2]. High sorption capacity results in low mobility [3, 4].

Sojka et al. [1] reported that PAM degradation occurs slowly in soils and by several different mechanisms. These include biotic and abiotic transformation such as chemical, photochemical, and biological processes as well as mechanical processes, such as tillage abrasion, freezing and thawing. First, abiotic processes break the polymer into progressively shorter segments. When polymer segments are reduced to 6 or 7 monomer units long, they are then utilized by soil microorganisms [1]. Overall, degradation rates in soil are estimated to be around 10% per year. Modest degradation was also reported by Wolter et al. [5] and Stahl et al. [6]. Soil microcosm experiments examining biodegradation rates of cross-linked PAM copolymer indicated degradation rates as high as 7% per 80 days. Chang et al. [7] examined the aerobic and anaerobic biodegradation of cationic-PAM and showed the polymer was subject to partial degradation under both conditions in laboratory inoculation–incubation tests. Measured O_2_ consumption under aerobic conditions, and gas production under anaerobic conditions indicated that the partial destruction of pendant cationic moieties occurred by ester hydrolysis, but the polymer’s backbone, which exclusively exhibits carbon–carbon bonds, remained essentially intact. Ester hydrolysis of the polymer side chain releases choline and anionic-PAM. Other authors focused on the investigation of the microbial processes. For example, Nakamiya and Kinoshita [8] isolated two bacterial strains from soil, *Enterobacter agglomerans* and *Azomonas macrocytogenes,* with the ability to degrade PAM. Both strains grew on a medium composed of 10 mg/mL PAM as the sole source of C and N. After 27 h incubation, about 20% of the total organic C in the initial medium was consumed and the average MW of PAM was reduced from 2 × 10^6^ to 0.5 × 10^6^ by microbial degradation. Fungi also have the ability to decompose PAM. Stahl et al. [6] investigated the biodegradation of two superabsorbent polymers (namely a crosslinked, insoluble polyacrylate and an insoluble polyacrylate/polyacrylamide copolymer) in soil by the white-rot fungus *Phanerochaete chrysosporium*. The polymers were both solubilized and mineralized by the fungus but solubilization and mineralization of the copolymer was much more rapid than that of the polyacrylate. Soil microbes poorly solubilized the polymers and were unable to mineralize either intact polymer. However, soil microbes worked in conjunction with the fungus during polymer degradation in soil, whereby the fungus solubilised the polymers and the soil microbes stimulated mineralization. Furthermore, soil microbes were able to significantly mineralize both polymers after solubilization by *P. chrysosporium* grown under conditions that produced fungal peroxidases or cellobiose dehydrogenase, or after solubilization by photochemically generated Fenton reagent. The results suggested that biodegradation of these polymers in soil was optimum under conditions that maximized solubilization. Wolter et al. [5] quantified the biological degradation of a ^14^C-labelled acrylamide/acrylic acid copolymer in an agricultural soil by two white rot fungi (*Pleurotus ostreatus* and *Dichomitus squalens*), a brown rot fungus (*Flammulina velutipes*) and a saprophytic soil fungus (*Agaricus bitorquis*) in soil microcosms. The highest mineralisation of the ^14^C-copolymer to ^14^CO_2_ was measured following the inoculation of the soil with *P. ostreatus* (8.8% of the initial radioactivity within 22 weeks).

Even though PAM has been reported to be non-toxic to the ecological system and modest transformation in soil has been reported by several authors there is still concern in connection with the risk to sludge-amended soils. Based on these concerns and the precautionary principle, which is one of the basic tenets of the German soil protection act, the German Fertilizer Ordinance DüMV of 5th December 2012 [9] introduced a trigger value for degradation of synthetic polymers of 20% in a 2-year period.

When determining the degradation potential of PAMs in soils or sludge-amended soils experimental challenges need to be addressed. Among others, there are significant problems in extracting PAMs from soil or sludge matrixes for quantification by conventional methods due to the degree of sorption of the polymer. Thus, the use of a radioactive polymer was the only option to follow the fate of PAMs. We mimicked the land-spreading of sludge using ^14^C-PAM to flocculate sludge, which was then applied to the lysimeter. The dewatered sludge was applied to undisturbed soils in outdoor lysimeters followed by agricultural treatment. Thus, degradability and leaching of PAM were studied at realistic outdoor exposure conditions and concentrations after applying ^14^C material using highly sensitive ^14^C detection techniques.

## Methods

### Test substance synthesis and characterization

#### Synthesis

360 MBq of the ^14^C-labelled monomer Acrylamide [2,3-^14^C] (Lot No. 101022, specific activity 2.60 MBq/mg, chemical purity > 99%) was purchased from ARC, and used for the polymerisation procedure. For synthesis, 0.0225 g Versenex 80 (10%, Ashland), 1.88 g ^14^C-acrylamide (50% in water), 3.15 g ADAME-QUAT ([2‐(acryloyloxy)ethyl]trimethyl-ammonium chloride, 80%, Ashland) were adjusted with H_2_SO_4_ to pH 4. As initiator, freshly prepared 0.055 g ABAH (2,2′-Azo-bis(2-amidinopropane) dihydrochloride, 10%, Ashland) was added, cooled to − 10 °C with dry ice/ethanol mixture and exposed to UV light for 1 h. The synthesis product was dried for 90 min at 90 °C and ground in a mill to < 1 mm.

#### Characterisation of ^14^C-polyacrylamide copolymer (PAM)

*Viscosity* Viscosity was determined by a Brookfield viscometer with UL-adapter at a concentration of 0.5% ^14^C-PAM and resulted in 760 cp at 1.0 rpm, and 670 cp at 2.5 rpm. Compared to the commercial product, which has an average molecular weight between 5 and 8 million Daltons, ^14^C-PAM was at the lower end of the specification, i.e., a molecular mass of 6 million Daltons.

*Monomer content* The monomer content was 2820 ppm measured by radio-HPLC which is in the range of commercial PAM, where all educts are below 1000 ppm.

^*14*^*C*-*Radioactivity* In total 3.6 g ^14^C-PAM with a total radioactivity of 339.3 MBq was produced. The specific radioactivity was 94.25 kBq/mg.

**Fig. 1:**
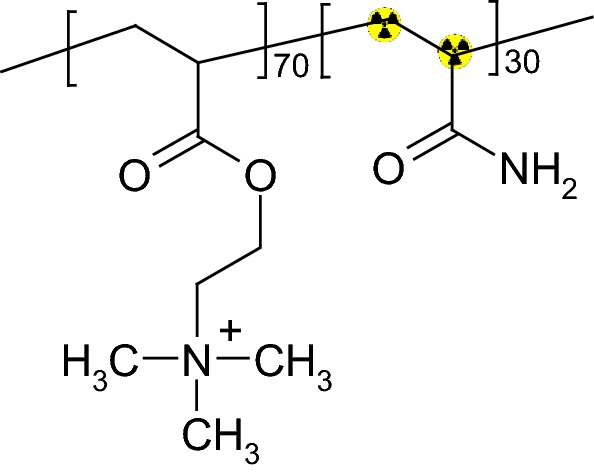
Chemical structure of ^14^C-PAM used in the study

*Chemical structure of* ^*14*^*C*-*PAM* The procedures described above yielded the chemical substance as presented in Fig. 1. It is important to note that the label is located on the polymer backbone of the 30 mol-% acrylamide repeating units. Hydrolysis or decarboxylation of any side chain, as well as mineralization of the carboxylic backbone were detectable in this study only by reduction of the molecular weight.

### Sludge flocculation with ^14^C-PAM

#### Flocculation procedure

3.5 g ^14^C-PAM (329.9 MBq) was dissolved in 1 L water for flocculation of digested sludge which was purchased from a local sewage treatment plant (Repetalstraße 421, 57439 Attendorn, Germany; average daily waste water amount 11,000 m^3^, 29,800 population equivalents). Optimal PAM concentration for flocculation was determined right before the flocculation procedure. Flocculation was achieved by dissolution of 3.5 g of ^14^C-polymer in 1 L water; flocculation of 105 L of sludge at 33.33 ppm in a 5 L beaker; slow manual mixing with a stirrer for 1 min; settling for 5 min and suction of the supernatant water. The solids were transferred to 1 L centrifuge jars, centrifuged at 1105×*g* for 5 min and the cake stored at 4 °C.

For a measured sludge dry solids concentration of 7.7 g/L, the flocculants dosage was 4.329 kg/TDS. Finally, 9247 g dewatered sludge with a dry matter content of 9.43%, corresponding to 872 g dry substance was produced. This was the test material (theoretical amount: 329.9 MBq/872 g dewatered sludge) used for the lysimeter experiments.

#### ^14^C mass balance and efficiency of flocculation process

During the flocculation procedure, some waste material containing ^14^C-labelled test material was produced. Significant amounts of ^14^C-material remained in the wastewater, and further amounts were adsorbed to the glassware used for the polymerization. In total, losses amounted to 92.46 MBq (28%). To determine the effective radioactivity in the treated sludge, 3 individual samples were taken from the sludge, dried and ground with sand at a known ratio to a uniform consistency. Data on combustion of 5 sub-samples each showed very good homogeneity, and the final radioactivity was determined to be 272.72 ± 16.2 kBq/g sludge dw corresponding to 237.42 MBq total radioactivity. The amount of 237.42 MBq was used as basis for further calculations, e.g., the application rate.

### Extraction of ^14^C-PAM flocculated sludge mixed with soil

#### Extraction with NaOH

Extraction efficiencies of the soil/sludge mixture using ZnCl_2_ or CaCl_2_-solution were below 40% and, furthermore, irreversible precipitation during the subsequent dialysis occurred. Instead, ^14^C-PAM was extracted from the soil by a NaOH-solution. Such treatment led to hydrolysis of the PAM side chains and partially destroyed the soil matrix while the C–C polymer chain (“backbone”) was stable. The sludge/soil mixtures were shaken for 24 h using 0.5 M and 1 M NaOH-solution. Extraction efficiencies were 120% aR (0.5 M NaOH) and 125% aR (1 M NaOH). An explanation of the high values might be that the water content of the sludge has changed slightly due to settling processes. Thus, initial radioactivity, which further calculations are referred to, will be slightly underestimated. More than 97% of the extracted radioactivity remained in solution during dialysis of the NaOH-extract.

#### Quantification of non-extractable residues after NaOH extraction

The extracted soil was dried and ground. 200 mg sub-samples were combusted by means of an oxidizer, evolved ^14^CO_2_ was trapped in NaOH-containing traps and subsequently quantified by liquid scintillation counting (LSC). LSC measurements were performed using a Packard Tri–Carb liquid scintillation analyzer or a Hidex 300 SL. Each sample was measured for 5 min. Computer–constructed quench curves, derived from a commercially available series of sealed quenched standards, automatically converted counts per minute (cpm) to decays per minute (dpm).

### GPC

The NaOH extract was dialyzed (nominal MWCO 12,000–14,000 Da) against water to neutralize it without increasing the salt load while keeping the polymer in solution. The pH after dialysis was approximately 7.5. An aliquot of 10 mL of the dialyzed sample was evaporated to 1 mL for subsequent gel permeation chromatography (GPC). Recovery after concentration was 92%. The polymer concentration in the sample (calculated on the basis of a specific activity of 94.25 kBq/mg) was 9000 Bq/mL = 95.5 mg/L. 100 µL of the concentrated extract was injected into the GPC. By collecting the eluent and analyzing for total radioactivity it was verified that no radioactivity was retained by the GPC-column.

For GPC a Dionex HPLC, equipped with a UV-detector (Dionex PDA 100, detection wavelength 230 nm) and a ^14^C-detector (Raytest Ramona Star, flow through, cell volume 200 µl) connected in series was used. The following set of GPC-columns (Polymer Standards Service GmbH, Mainz, Germany) were used:

Guard pre-column PSS MCX 10 µ; 8.0 × 50 mm.

Column PSS MCX 10^3^ Å; 10 µ; 8.0 × 300 mm.

Column PSS MCX 10^5^ Å; 10 µ; 8.0 × 300 mm.

An isocratic eluent consisting of 0.07 mol/L Na_2_HPO_4_ with 10 ppm H_3_PO_4_ in deionized water was run at 1 mL/min and 25 °C. Injection volume was 100 µl.

**Fig. 2:**
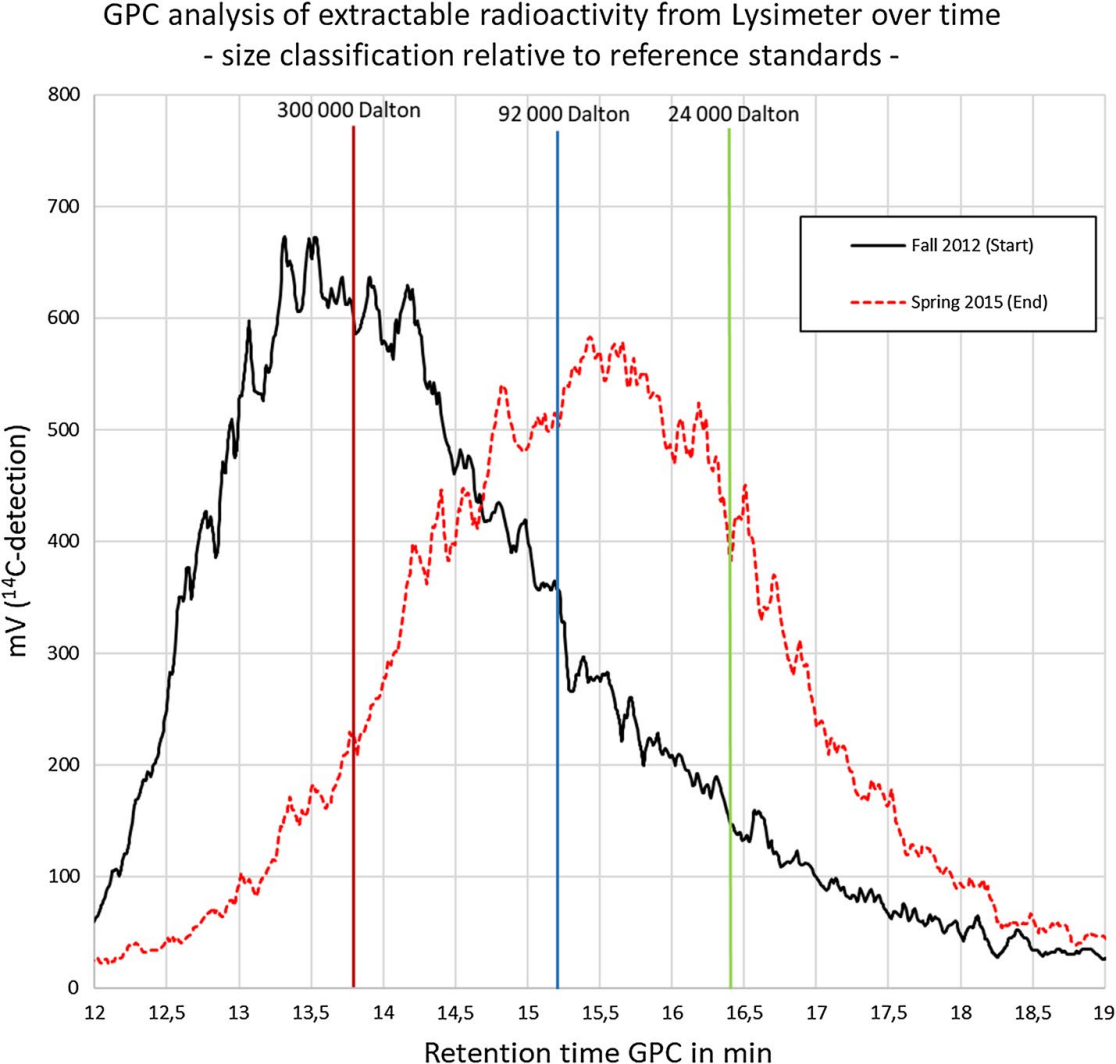
Lysimeter-extract at test start and test end with standard retention times. Vertical lines = retention time of molecular weight reference standards: red: SaSt140317-AS (300,000 Da), 13.8 min; blue: SaSt140317-BS (92,000 Da), 15.2 min; green: SaSt140317-CS (24,000 Da), 16.4 min

**Fig. 3:**
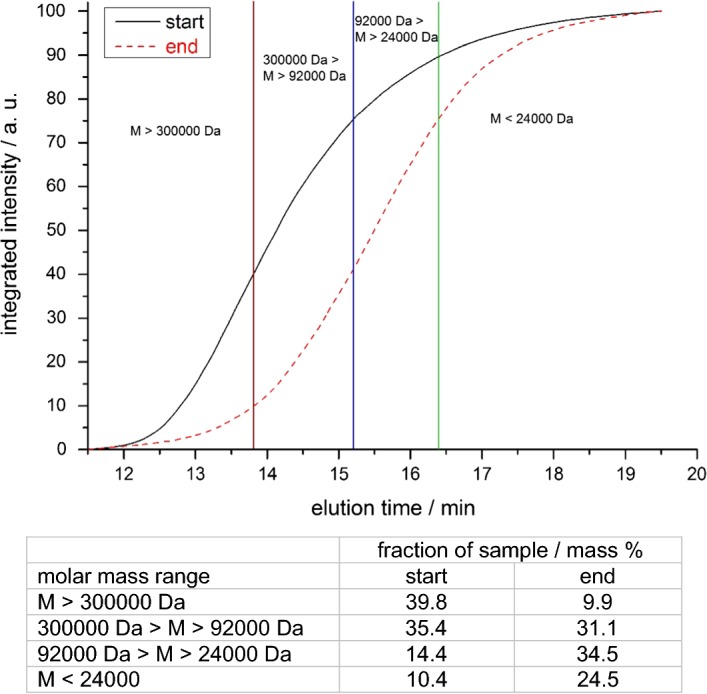
Molar mass distribution of the lysimeter extract at test start and test end relative to molecular weight reference standards

Due to series connection of UV-detector and ^14^C-detector there was a signal delay of the ^14^C-signal relative to the UV signal of about 0.5 min. Thus, the retention times of the non-radioactive GPC-standards had to be corrected accordingly to compare with the retention times measured with ^14^C detection. A detailed description of the reference standards is shown as Additional file 1 (document: additional information RAFT polymerization.pdf). Figure 2 shows the result of the measurement of the soil extracts from start and end of the Lysimeter experiment and the corrected retention time of the molecular weight reference standards. Based on the measurements a molar mass distribution within the soil extracts was calculated and transformed into the diagram shown in Fig. 3.

### Outdoor lysimeter system

#### Description of test system

**Fig. 4:**
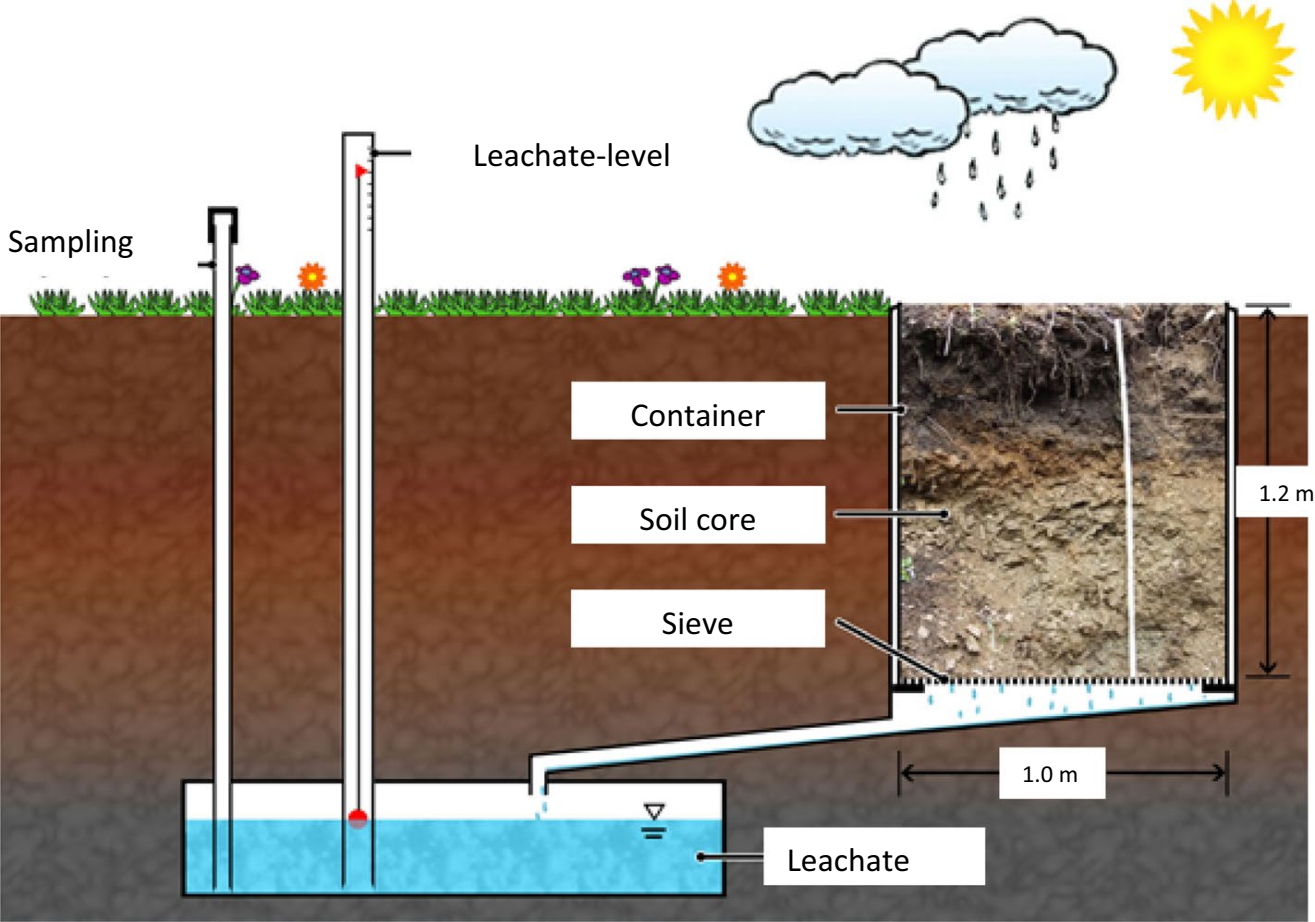
Scheme of outdoor lysimeter

The experiment was conducted in a lysimeter (see Fig. 4) containing an undisturbed soil core from a field used for agriculture. The soil core had a height of 1.0 m and a surface area of 1.08 m^2^. A sieve plate at the bottom of the lysimeter allowed leachate to run out. The leachate was collected in a subsurface tank and sampled as soon as the leachate reached a certain level in the tank. The duration of the experiment was 3 years. A loamy sand soil (named RefeSol 01-A soil) was used, whose characteristics are given in Additional file 2: Table S1.

#### Dose calculation and application

A maximum PAM concentration of 25 kg PAM/ha corresponding to 2.5 g PAM/m^2^ was the basis to calculate the amount of ^14^C-PAM to be dosed. A rate of 2.5 g ^14^C-PAM corresponds to 228 MBq in 838.5 g sludge dw/m^2^. 179.7 MBq ^14^C-PAM corresponding to 2.0 g ^14^C-PAM in 660 g sludge dw was applied to 1 m^2^ lysimeter surface area.

On 24th October 2012, an outdoor lysimeter was dosed by thoroughly mixing the flocculated digested sludge with 5 cm of the top soil layer. After mixing, the soil/sludge mixture was evenly distributed on the lysimeter surface. At 1 DAT, soil/sludge samples were taken at 5 different sampling points from the 0 to 5 cm top layer and analyzed for radioactivity content by combustion and subsequent radio-assaying. After that sampling, the lysimeter and surrounding area were sown with wheat (SW Kadrilj) according to GAP.

#### In-life phase

Wheat (SW Kadrilj) was grown during the season 2012/2013 and no further treatment except fertilization with mineral fertilizer according to GAP was done. Thereafter, the following cultures were sown and harvested from the lysimeter: wheat (SW Kadrilj, sown April 2013), spinach (Emilia, sown May 2014), mustard seed (“green manure”, sown April 2015), wheat (SW Kadrilj, sown June 2015).

Leachate volumes are given in Additional file 2: Table S2. As no additional irrigation was performed at any time during the study, the leachate is due to natural rain events only. In total, 2465 L of leachate were collected within almost exactly 3 years. This is in line with the expected average precipitation of about 800 mm/year for the test site Schmallenberg, Germany (51.1526N, 8.2908E).

#### Sampling

The second soil sampling of the lysimeter was performed on 24th April 2013 (“spring 2013”), 6 months after application. Any earlier sampling was not possible due to the long winter season resulting in frozen top soil. Further samplings were performed on 2nd October 2013 (“fall 2013”), 5th May 2014 (“spring 2014”), 28th October 2014 (“fall 2014”), 5th May 2015 (“spring 2015”), and 26th October 2015 (“fall 2015”). At the first sampling, the top 0–5 cm layer only was sampled. At all further samplings, the 5–10 cm layer was additionally sampled. Furthermore, at the last sampling, core samples up to a 50 cm soil depth were taken. These soil cores were divided into 10 cm segments each, and analyzed for ^14^C-radioactivity to examine vertical movement of the applied material. At each sampling, soil was taken at 5 individual positions by means of an auger.

Quantification of ^14^C-radioactivity in soil samples was done by homogenization of samples, subsequent combustion of sub-samples, trapping of evolved ^14^CO_2_ and LSC-measurement of trapping solution.

The amount of leached radioactivity was monitored by radio-counting aliquots of each of the sampled leachates.

Harvested plants were individually separated into their different parts, cut into pieces, homogenized and milled to uniform consistency. Aliquots were combusted with subsequent LSC-analysis.

### Calculation of transformation half-life times and DTx-values

PAMs were analyzed based on the recommendation of the FOrum for the Co-ordination of pesticide fate models and their USe (FOCUS) degradation kinetics [10, 11]. In addition to the standard kinetics (SFO = single first order) FOCUS recommends three bi-phasic kinetics, which are often more suitable to describe the fate of substances than the traditional single first order degradation. The additional kinetics are: HS (Hockey stick), DFOP (Double first order in parallel), and FOMC (First order multi compartment). The use of these kinetic models allowed calculation of the time needed for the disappearance of 50% (DT_50_-value) and 90% (DT_90_-value) of the compound(s) under consideration. Furthermore, the *χ*^2^-value indicated the robustness of the calculation.

By means of the CAKE-software tool [12], DT_50_-values and DT_90_-values were calculated from the loss of radioactivity in the top 0–10 cm lysimeter layer. Calculations were conducted using all the kinetic models and the *χ*^2^-values of all the kinetic models were compared. Furthermore, a visual check of the graphs of all models was performed. From both comparisons it was obvious, that none of the kinetic models was better than the SFO-model.

Equation (2), which is obtained from Eq. (1), was used to determine *C*(*t*_X_) at various *t*_X_–intervals within a 0–10 years period.

1 $${\text{DT}}_{ 50} = {\text{ ln 2}}/k$$

2 $${ \ln }\left( {C\left( {t_{\text{x}} } \right)/C\left( 0 \right)} \right)\, = \, - \,k\, \times \,t_{\text{x}}$$

## Results and discussion

### Radioactivity distribution in soil layers and mass balance

To determine the total radioactivity in the top soil layer, samples from the 0–5 cm top layer were taken once the sludge had been incorporated into that layer. For the following periods, 0–5 and 5–10 cm layers were sampled separately. However, tillage resulted in an intense physical mixing of both layers, which in turn reduced the significance of such differentiated sampling. Consequently, results for the 0–5 and 5–10 cm layers were re-calculated and reported as a single 0–10 cm layer. These data served as input for further assessment and calculations of kinetics.

**Fig. 5:**
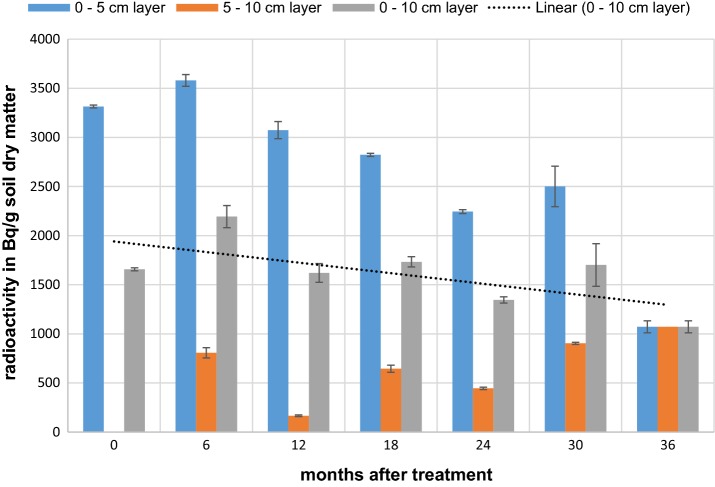
Concentration of radioactivity (Bq/g soil dw) in soil layers at various sampling days after treatment. Dotted line: linear presentation of results for the 0–10 cm layer

At 1 DAT, a concentration of 3315 Bq/g soil dw was measured by combustion analysis for the 0–5 cm layer, which is equivalent to 1657 Bg/g soil dw in the 0–10 cm layer. This value serves as 100%-basis (1657 Bq/g soil dw ≡ 100% aR) for calculating the actual amount of radioactivity in relation to the applied amount. Detailed information on both measured (mean of 3 replicates) and calculated data are presented in Additional file 2: Table S3. Figure 5 presents the entire set of data, i.e., those obtained for the 0–5 cm layer, 5–10 cm layer, 0–10 cm layer (calculated) as well as a linear presentation of the results for the 0–10 cm layer.

Already from such a simple linear presentation of the results for the 0–10 cm layer a modest but constant decrease of radioactivity within that layer was observed.

**Fig. 6:**
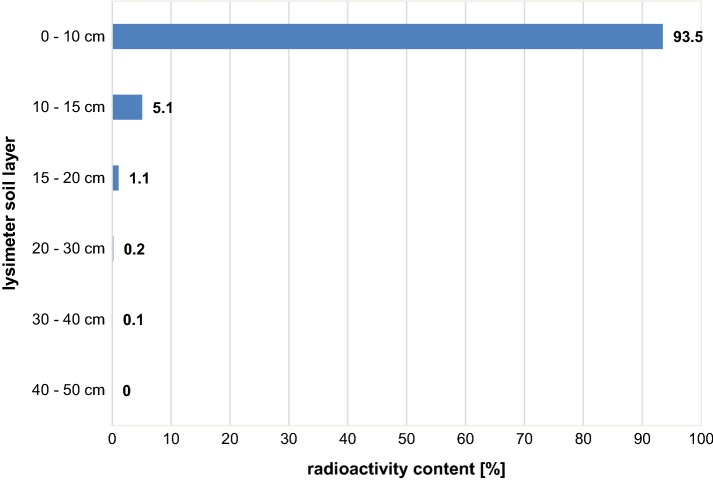
Vertical radioactivity profile 3 years after treatment. Results in (% of total amount present at 1095 DAT)

To accomplish mass balance, a radioactivity profile down to a soil depth of 50 cm and at 1095 DAT (termination of the study 3 years after treatment) was determined. Five soil cores were taken and separated into 0–10, 10–15, 15–20, 20–30, 30–40, and 40–50 cm segments. Figure 6 shows the vertical radioactivity profile. Results are presented as % of the total amount still being present in the soil at 1095 DAT.

It is obvious, that ^14^C-PAM is immobile in soil as only 6.5% was translocated beyond the top 10 cm. Only 0.1% was found in the 30–40 cm layer. The predominant but not exclusive reason for such movement might be a mechanical soil treatment during sowing and harvest rather than translocation by leaching. This conclusion is supported by the fact that no radioactivity was detected in any of the leachate samples. These results are in full agreement with those published by Nadler et al. [3]. The authors found that PAM remained stable at the original application depth even 10 months after application and with 720 mm of additional water application. Sojka et al. [4] reported, that due to its high sorptive ability and low mobility, high molecular weight PAM does not move more than a few centimetres from its point of entry into the soil.

Combustion analysis of the homogenized plants proved that no radioactivity was present in straw, grain or spinach. Thus, no plant uptake is likely to occur.

Summarizing all observations and facts, a reasonable explanation for the decreasing radioactivity content in the upper 10 cm soil layer (Fig. 5) is a slow degradation with subsequent mineralization and thus release of ^14^CO_2_ from the lysimeter. To confirm this hypothesis a lab experiment would be needed with specific setup to detect very slow ^14^CO_2_ formation.

### Extractable and non-extractable residues

To characterize radioactivity which is present in the 5 cm top soil layer, soil samples were extracted. The most effective non-matrix-destructive extraction method was that using 5 M ZnCl_2_-solution. However, the extraction resulted in about 75% NER directly after mixing flocculated sludge and soil. Consequently, this approach was not followed. Though being a destructive method, extraction using 1 M NaOH was finally chosen as it recovered maximum radioactivity for subsequent analyses. Table 1 presents the radioactivity distribution in extracts and remaining soil residues at various days after treatment.

**Table 1:** Distribution of radioactivity in 0–5 cm top soil layer (% aR) after 1 M NaOH extraction

	Percent of applied radioactivity by days after treatment					
	1	183	365	578	730	913
Extractable	98.1 ± 1.6	102.6 ± 3.3	83.4 ± 3.7	79.6 ± 1.3	62.9 ± 2.3	63.9 ± 1.1
Non-extractable residues	8.0 ± 1.4	11.3 ± 1.4	10.8 ± 0.9	10.8 ± 0.8	8.4 ± 1.2	8.7 ± 1.1
Recovery^a^	106.1	113.9	94.2	90.4	71.3	72.6

^a^Recovery is calculated by summing up amounts in extracts and non-extractables

The data presented in Table 1 show a clear trend. The extractable radioactivity decreases continuously and the non-extractable residues remain constant at around 10% of the applied radioactivity.

In OECD Guideline 307 [13] it is stated in Annex 1, Definitions, that “the extraction method must not substantially change the compounds themselves or the structure of the matrix”. As the extraction method used in the present study is a matrix-destructive extraction, the data shown in Table 1 overestimate the extractable fraction and underestimate the amount of non-extractable residues. According to FOCUS [10, 11], NERs should be regarded as transformation products, and thus, the amount of transformation products might be underestimated when following this definition.

### GPC-analyses of soil extracts

**Fig. 7:**
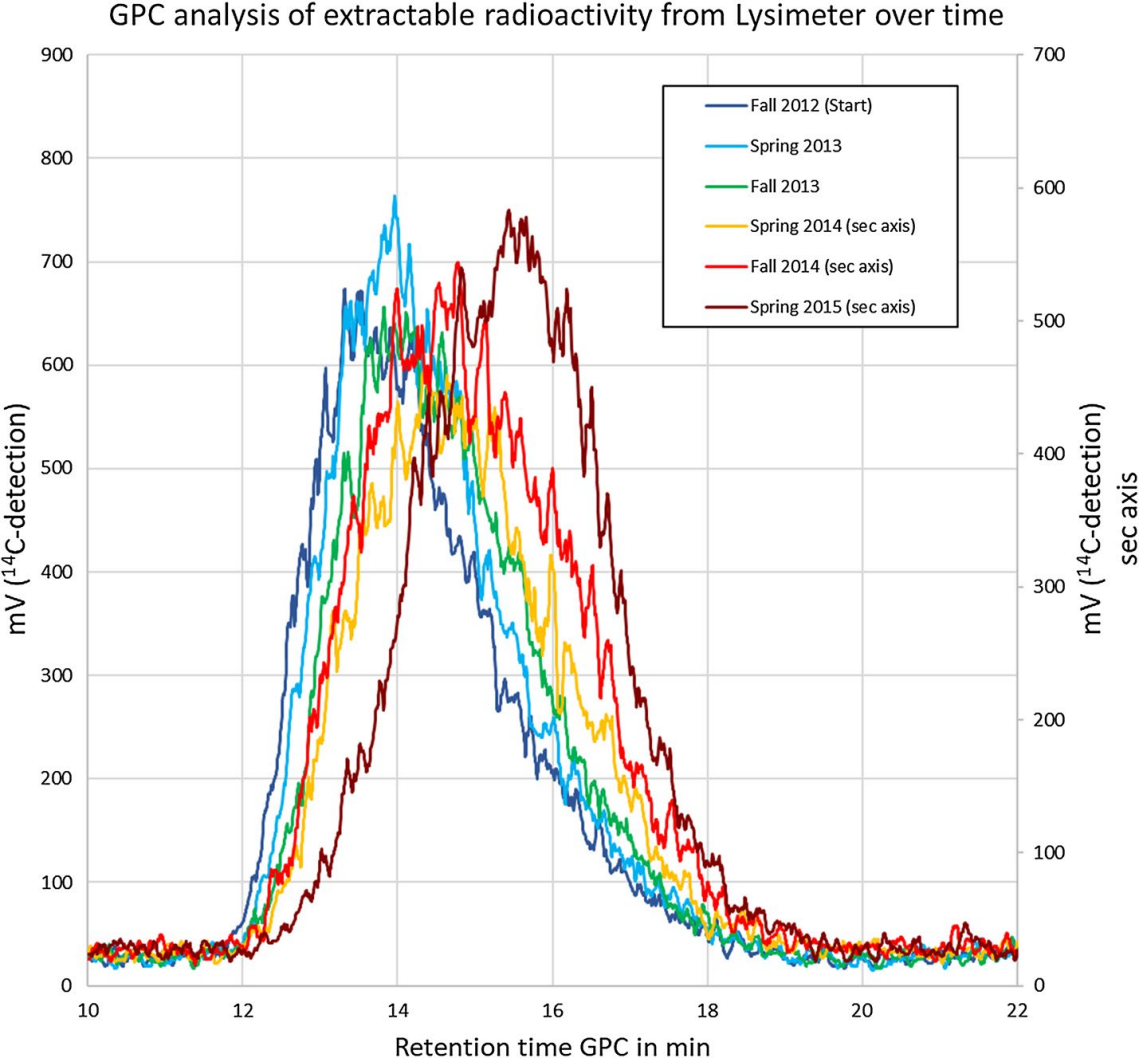
Overlay of GPC-signals from lysimeter soil extracts obtained by radio-detection

**Fig. 8:**
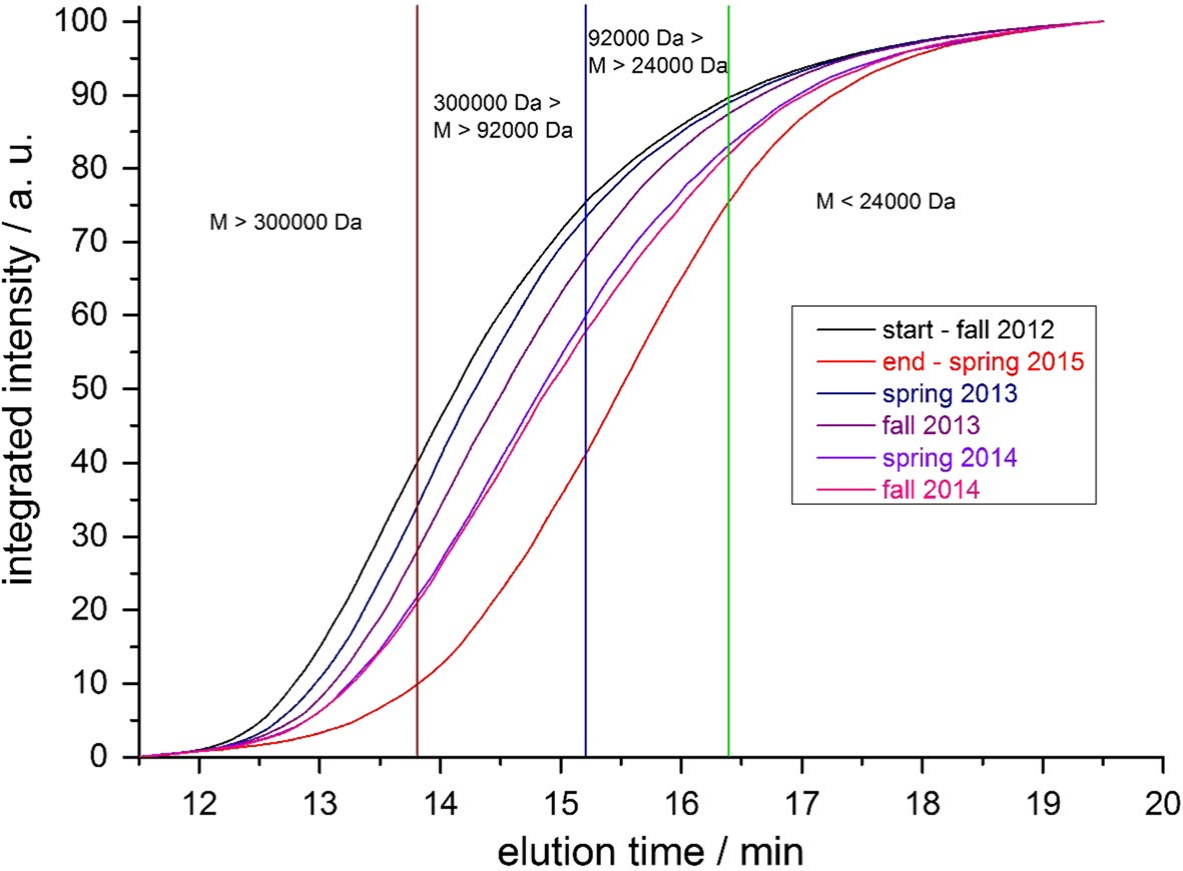
Development of molar mass distribution of the lysimeter soil extracts with time

After clean-up (see “GPC”) of NaOH-extractable radioactivity from the 5 cm top soil layer, extracts from all soil samples were analyzed by GPC. The GPC-chromatogram shows broad signals which is due to the typical molar mass distribution of a synthetic polymer prepared by free radical polymerization. Figure 7 shows an overlay of all chromatograms. Though it is difficult to visualize, it clearly shows a shift to longer signal retention times for the later sampling dates. This means that the longer the period after treatment, the signal retention time increases, which equates to smaller average molar masses of the extracted polymer. To better visualize and quantify this result, the molar mass distribution has been calculated for each sample based on the molar mass reference standards shown in Fig. 2. Table 2 gives an overview on the molar mass distribution. It shows a constant increase of the fraction below 24,000 Da and at the same time a decrease of the fraction above 300,000 Da. In other words, over a time interval of 913 days a significant and continuous reduction of the average molar mass of the radioactive PAM was observed. Figure 8 visualize the change of the molar mass distribution.

**Table 2:** Molar mass distribution in lysimeter soil extracts determined by GPC based on reference standards

Sample	M > 300,000 Da	300,000 Da > M > 92,000 Da	92,000 Da > M > 24,000 Da	M < 24,000 Da
1 DAT (Start)	39.8	35.4	14.4	10.4
183 DAT (spring 2013)	34.2	39.0	15.7	11.1
365 DAT (fall 2013)	28.2	39.6	19.7	12.5
578 DAT (spring 2014)	21.7	38.2	23.2	16.9
730 DAT (fall 2014)	20.9	36.8	24.1	18.2
913 DAT (spring 2015)	9.9	31.1	34.5	24.5

Data in % of total signal area

### Degradation kinetics

**Fig. 9:**
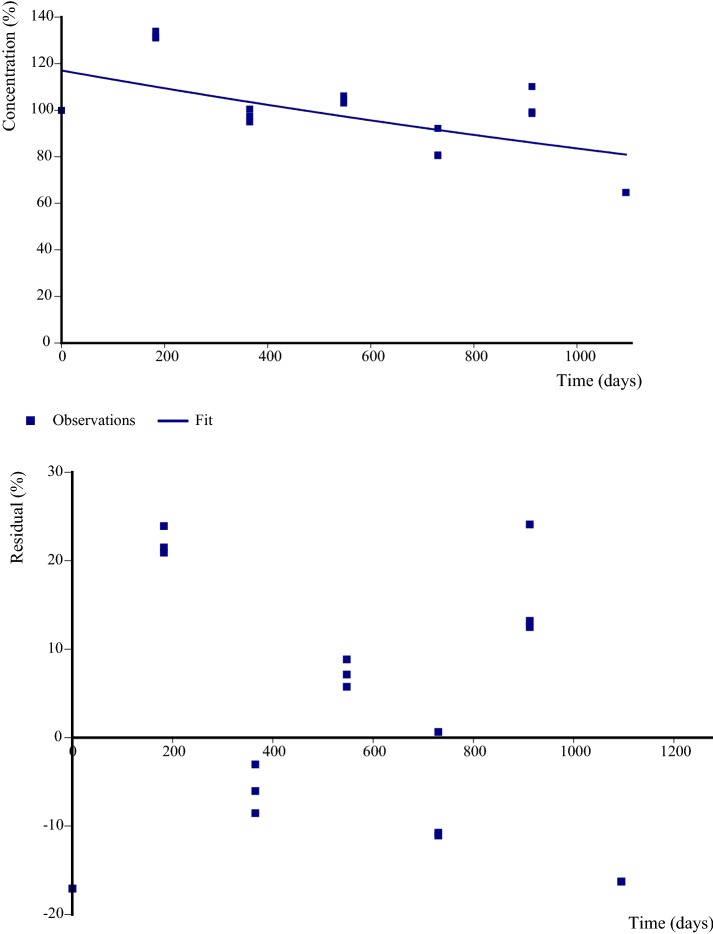
CAKE model evaluation plot for disappearance of radioactivity (% aR) from the top 0–10 cm lysimeter layer. Observations and fitted model (above), residuals (below). Kinetic model: single first order. Prob > *t*: 0.001. Parent rate constant: 3.5e−004/day, 3 replicates

“Ultimate biodegradation” results in, but is not limited to the production of CO_2_ [14, 15]. It might also lead to incorporation into microbial biomass or carbonates which would be detected as NER. On the other hand NER can also be association of the parent chemical or breakdown product with the soil matrix. However, to calculate the degradation kinetics of ^14^C-PAM in an outdoor lysimeter, data on ^14^C-radioactivity content in the upper 10 cm soil layer only are used as an input. In the end, formation of ^14^CO_2_ is the only transformation process which is taken into account. Such an approach can be regarded as conservative with respect to ^14^C-PAM degradability as neither the formation of non-extractable residues nor the reduction of the initial molar mass are included transformation processes (Fig. 9).

Additional file 2: Table S4 shows the input data used to calculate the degradation kinetics of ^14^C-PAM in the lysimeter. First, kinetic analyses were performed using all available kinetic models, namely single first order (SFO), first order multi compartment (FOMC), hockey stick (HS), and double first order in parallel (DFOP). Selection of the most appropriate kinetic model was based on the criteria, that according to [10, 11] ideally, the error value at which the *χ*^2^-test is passed by the best-fit model (SFO, FOMC or DFOP) should be below 15%, and the fit must be visually acceptable.

The disappearance of ^14^C-PAM from the 10 cm top layer of an outdoor lysimeter was described by the SFO kinetic model as the *χ*^2^—value is 12.0 and the prob > *t* value is 0.001. This indicated that the rate is considered significantly different from zero as the probability is smaller than 0.05, i.e., considering a 5% significance level.

The DT_X_-values are calculated to be:

$${\text{DT}}_{ 50} \, = \, 2.0\, \times \, 10^{ 3} \,{\text{days}}\, = \, 5. 5 {\text{ a}}$$

$${\text{DT}}_{ 90} \, = \, 6. 6 5\, \times \, 10^{ 3} \,{\text{days}}\, = \, 1 8. 2 {\text{ a}}.$$

Further statistical information is presented in Table 3.

**Table 3:** Detailed results of kinetic modelling

	k (parent)	All data
Value/day	0.00035	
Prob > *t*	0.001	
90% confidence interval		
Lower	1.79 × 10^−4^	
Upper	5.15 × 10^−4^	
95% confidence interval		
Lower	1.43 × 10^−4^	
Upper	0.001	
*χ* ^2^		
Value		12
Degree of freedom		5
Additional statistics		
*r*^2^ (Obs v. pred)		0.4191
Efficiency		0.4188

**Fig. 10:**
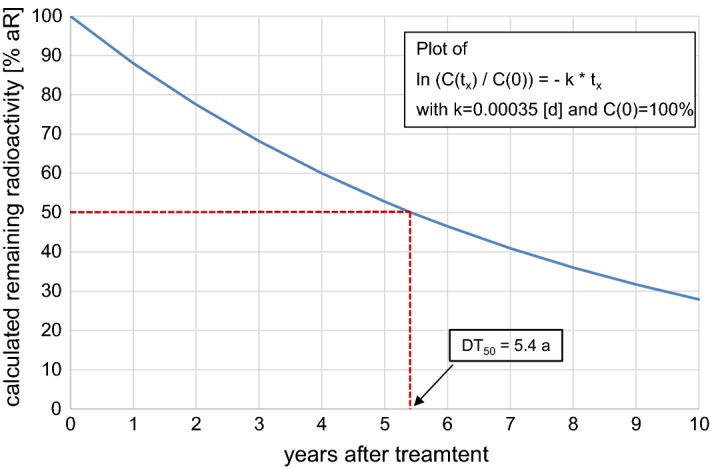
Projected decrease of remaining radioactivity (% aR) in the 10 cm top layer within 10 years after treatment

To estimate the radioactivity content *C*(*t*_X_) (% aR) being present at various intervals t_X_ after treatment, the equation ln $\left( {C\left( {t_{\text{x}} } \right)/C\left( 0 \right)} \right)\, = \, - \,k\, \times \,t_{\text{x}}$ was used. *C*(0) is equal to 100 (% aR), and *k* is equal to 0.00035/day. Additional file 2: Table S5 presents the detailed results, and Fig. 10 shows the calculated decrease of remaining radioactivity in the 10 cm top layer within 10 years after treatment.

It can be concluded that in a conservative evaluation of the experimental data from the outdoor simulation approach a kinetic rate constant for ultimate degradation can be obtained at a confidence level accepted in pesticide regulation. Based on that rate constant an ultimate degradation of 22.5% ^14^C-PAM within 2 years after application is obtained under outdoor conditions. This is in agreement with the summarized results reported by Sojka et al. [1], where degradation rates in soil were estimated to be around 10% per year.

Furthermore, the trigger value for degradation of synthetic polymers of 20% in a 2-year period, which was introduced by the German Fertilizer Ordinance DüMV of 5th December 2012 [9] is met.

## Conclusions

This project demonstrated that the synthetic cationic polyacrylamide copolymers (PAMs) incorporating C–C-bonds in the main chain slowly degrade in soil after land-spreading as a component of MWWTP sludge. Even in a conservative evaluation which considered only disappearance of radioactivity, corresponding presumably to the emission of carbon dioxide as mineralization product, the degradation rate was greater than 20% within 2 years which complies with the requirements of German Fertilizer Ordinance. Degradation was not exclusively mineralization, and therefore, the breakdown of the polymer backbone and subsequent integration into the soil matrix have to be taken into account when determining the fate of the polymer.

## Additional files

**Additional file 1.** RAFT polymerisation.

**Additional file 2: Table S1.** Soil characteristics of RefeSol 01-A. **Table S2.** Amount of leachate collected in the course of the lysimeter experiment. **Table S3.** Measured and calculated data for radioactivity content in the upper soil layers. **Table S4.** Input data (% aR) for calculation of kinetic parameters by means of CAKE. **Table S5.** Ultimate degradation in the top 10 cm layer calculated based on experimental data.

## Abbreviations

% aR: % of applied radioactivity
Bq: becquerel
cp: centipoise
DAT: days after treatment
DFOP: first order in parallel
TDS: tons dry solid
dw: dry weight
FOCUS: FOrum for the Co-ordination of pesticide fate models and their USe
FOMC: first order multi compartment
GAP: good agricultural practice
GPC: gel permeation chromatography
HS: hockey stick
PAM: cationic polyacrylamide copolymer
SFO: single first order
