# Supplementary material for: Cationic polyacrylamide copolymers (PAMs): environmental half life determination in sludge-treated soil
Source: Environ Sci Eur. 2018 May 18;30(1):16. doi: 10.1186/s12302-018-0143-3 (PMC5959997; doi:10.1186/s12302-018-0143-3)
Supplement: Supplementary file 1 — Additional file 1. RAFT polymerisation. [file 12302_2018_143_MOESM1_ESM.pdf]

Additional Information: RAFT method

Preparation of Poly(acrylic acid-co-cholin acrylate)s Utilising the RAFT Method and Characterisation of the Products

A series of poly(acrylic acid-co-choline acrylate)s were synthesised by the reversible addition-fragmentation chain transfer (RAFT) method. The ratio of constituents was chosen in order to emulate the final polymer composition of the material used in the study after being subjected to the basic extraction procedure. The compositions were verified by ^{1}H-NMR spectroscopy, and molar masses were determined by end group analyses, using ^{1}H-NMR spectroscopy, where possible, and UV/Vis spectroscopy. Furthermore, information was obtained about relative molar mass distributions by gel permeation chromatography.

Determination of Number Average Molar Masses

The statistical copolymerisation of acrylic acid and choline acrylate was performed in controlled conditions utilising a RAFT reagent. Details of the synthesis are given below. The resulting polymers (batch numbers SaSt140317AS to SaSt140317CS) were characterised by ^{1}H-NMR and UV/Vis spectroscopy. These spectra were used for end group analysis to determine the actual degree of polymerisation $\overline{P}_n$ and the number average molar mass values $\overline{M}_n$ of the polymers, as described in detail in the following. The integrals of the ^{1}H-NMR signals at 1.3 to 2.8 ppm (corresponding to the 3 hydrogen atoms of the polymer main chain) and the signals in the region of aromatic protons (7.0 to 9.0 ppm, corresponding to all ten hydrogen atoms of the RAFT reagent) were used to calculate the number average molar mass of the poly(acrylic acid-co-cholin acrylate)s SaSt140317AS, SaSt140317BS and SaSt140317CS.

Using these signals, $\overline{P}_n$ can be calculated according to:

$$\overline{P}_n = \frac{10 \cdot Int_{1.3 to 2.8 ppm}}{3 \cdot Int_{7.0 to 9.0 ppm}} \qquad (1)$$

For a poly(acrylic acid-co-cholin acrylate) of the chosen composition, the average molar mass M_{repeat} of the repeat unit is 73.28 g·mol^{-1}. Hence, the number average molar masses $\overline{M}_n$ are calculated according to:

$$\overline{M}_n = M_{repeat} \cdot \overline{P}_n \qquad (2)$$

The signals of the RAFT reagent are small relative to the signals of the polymer main chain, and become increasingly smaller with increasing molar mass. For the copolymer with the highest molar mass, signals assigned to the RAFT reagent are not detectable within the limits of the method. Hence, the molar mass determination based on ^{1}H-NMR signals was only applied to the two polymers with lower molar masses.

For SaSt140317BS:

$$\overline{P}_n = \frac{10 \cdot 300}{3 \cdot 1.37} \approx 730\,,\ \overline{M}_n \approx 73.3 \cdot 730 \approx 54000$$

For SaSt140317CS:

$$\overline{P}_n = \frac{10 \cdot 300}{3 \cdot 3.60} \approx 278\,,\ \overline{M}_n \approx 73.3 \cdot 278 \approx 20000$$

As an alternative method for the determination of $\overline{M}_n$ values, UV/Vis spectroscopy was employed.

Additional Information: RAFT method

Fundamental prerequisite for the determination of number average molar masses by UV/Vis spectroscopy is the existence of a wavelength range, in which only the RAFT group is absorbing. In this case, the prerequisite is fulfilled for the band with a maximum at 302 nm reasonably well. At 251 nm, there is still a noticeable contribution of the band, which can be assigned to the carbonyl group of poly(acrylic acid) (see spectrum in fig. 1). Therefore, only the absorbance data at 302 nm were taken into account for the end group analyses based on UV/Vis spectroscopy. Furthermore, these analyses are based on the assumption that the extinction coefficients of the RAFT groups in the polymers are close to the extinction coefficients ε of the RAFT reagent alone. Another prerequisite is the preservation of end groups in the polymer chains. Undesired chain termination and chain transfer reactions will lead to an artificially high $\overline{M}_n$ value.

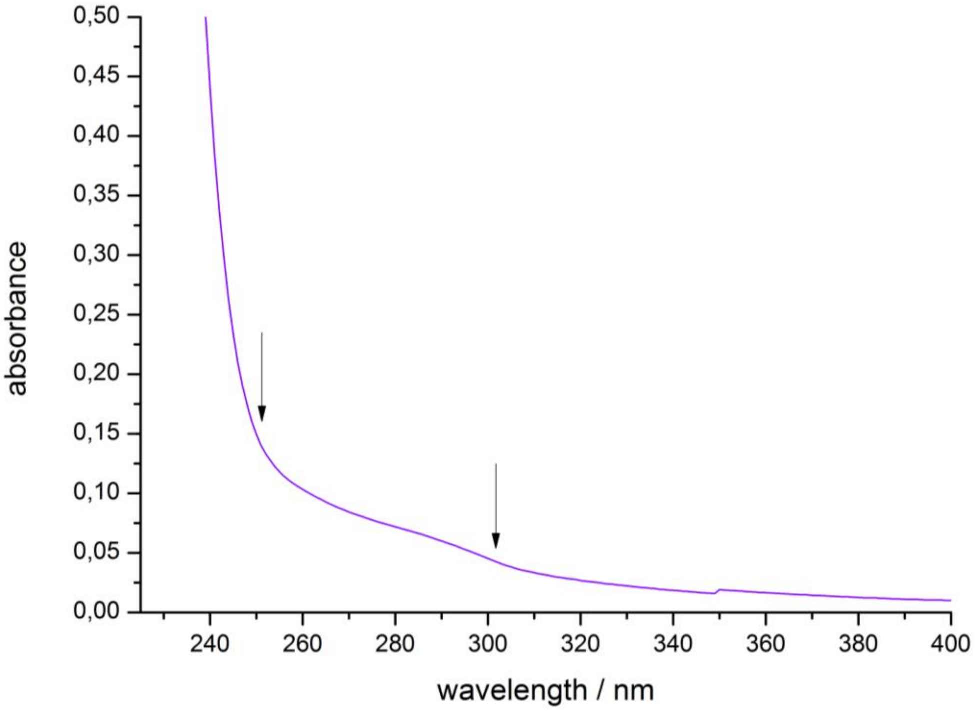

Fig. 1: UV spectrum of poly(acrylic acid) in high concentration in water. The arrows designate the positions of the maxima in the UV spectrum of the RAFT reagent, which is used in the project.

If polymer bearing RAFT groups is dissolved in a defined concentration $c_{Polymer}$, the concentration of RAFT groups c_{RAFT} in the solution can be calculated from the measured absorbance A according to:

$$c_{RAFT} = \frac{A}{\varepsilon \cdot d} \qquad (3)$$

d: length of the optical path in the solution,

ε: extinction coefficient of the RAFT reagent

Once this value is determined, the concentration of polymer repeat units in the solution can be calculated. The molar ratio of polymer repeat units to RAFT groups is the number average degree of polymerisation of the polymer.

$$c_{RAFT}\left[\frac{mol}{l}\right] \cdot M_{RAFT} = c_{RAFT}\left[\frac{g}{l}\right] \qquad (4)$$

$$c_{Polymer}\left[\frac{g}{l}\right] - c_{RAFT}\left[\frac{g}{l}\right] = c_{repeat}\left[\frac{g}{l}\right] \qquad (5)$$

Additional Information: RAFT method

$$\frac{c_{repeat}\left[\frac{g}{l}\right]}{M_{repeat}} = c_{repeat}\left[\frac{mol}{l}\right] \qquad (6)$$

$$\frac{c_{repeat}\left[\frac{mol}{l}\right]}{c_{RAFT}\left[\frac{mol}{l}\right]} = \bar{P}_n \qquad (7)$$

Results are summarised below, detailed calculations are given in the appended data sheet.

For SaSt140317AS:

Plot of absorbance versus polymer concentration at 302 nm:

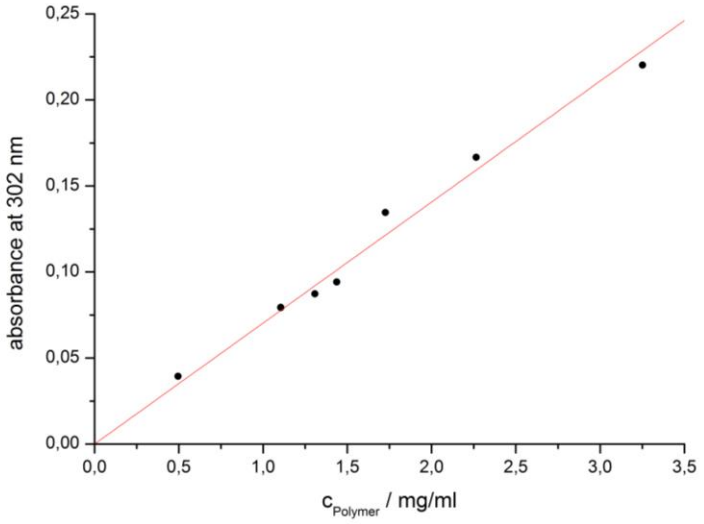

$\varepsilon_{302\ nm}$ = 21079 l·mol^{-1}·cm^{-1}

$\bar{P}_n \approx 4025$, $\bar{M}_n \approx 296000$

Additional Information: RAFT method

For SaSt140317BS:

Plot of absorbance versus polymer concentration at 302 nm:

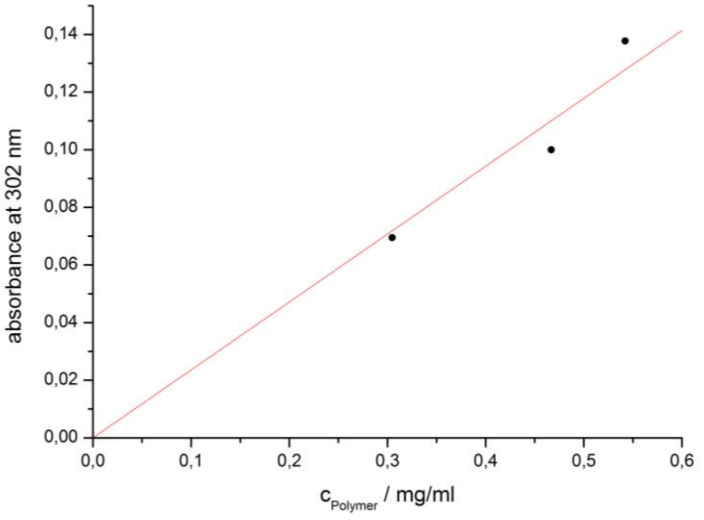

$\varepsilon_{302\ nm}$ = 21079 $l \cdot mol^{-1} \cdot cm^{-1}$

$\overline{P}_n \approx 1242$, $\overline{M}_n \approx 92000$ (detailed data see appendix).

For SaSt140317CS:

Plot of absorbance versus polymer concentration at 302 nm:

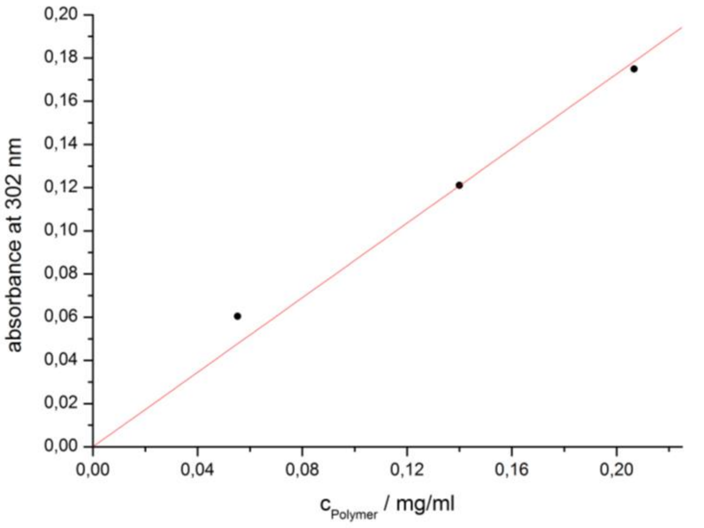

$\varepsilon_{302\ nm}$ = 21079 $l \cdot mol^{-1} \cdot cm^{-1}$

$\overline{P}_n \approx 309$, $\overline{M}_n \approx 23000$ (detailed data see appendix).

Additional Information: RAFT method

Averaging of values derived by ^{1}H-NMR and UV/Vis spectroscopy

Where possible, the calculations based on ^{1}H-NMR and UV/Vis were averaged.

Polymer no.	M_n by ^{1}H-NMR	M_n by UV/Vis	Overall averaged M_n
SaSt140317CS	20000	23000	21500
SaSt140317BS	54000	92000	73000
SaSt140317AS	-	296000	-

The overall averaged M_n values have a reasonable linear correlation with the peak masses of the GPC chromatograms (see Fig. 3). However, it must be remarked that the value of polymer does not fit in as well as the other values. The molar mass value as determined by ^{1}H-NMR would fit better for this polymer.

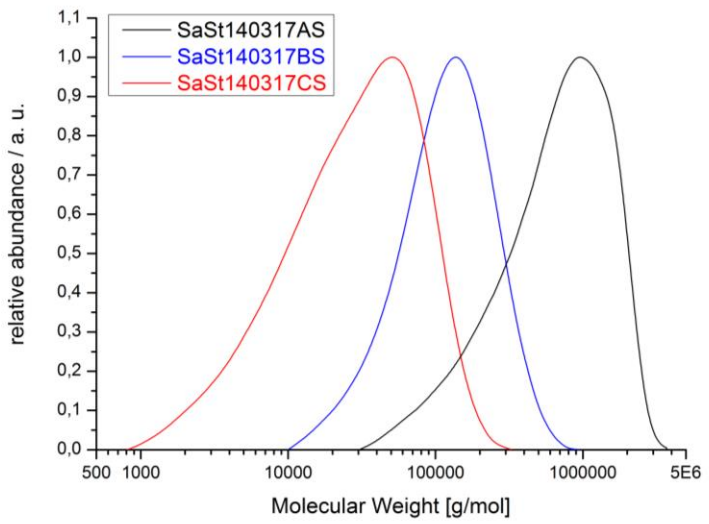

Fig 2: Chromatograms of three poly(acrylamide-co-cholin acrylate)s synthesised by the RAFT method.

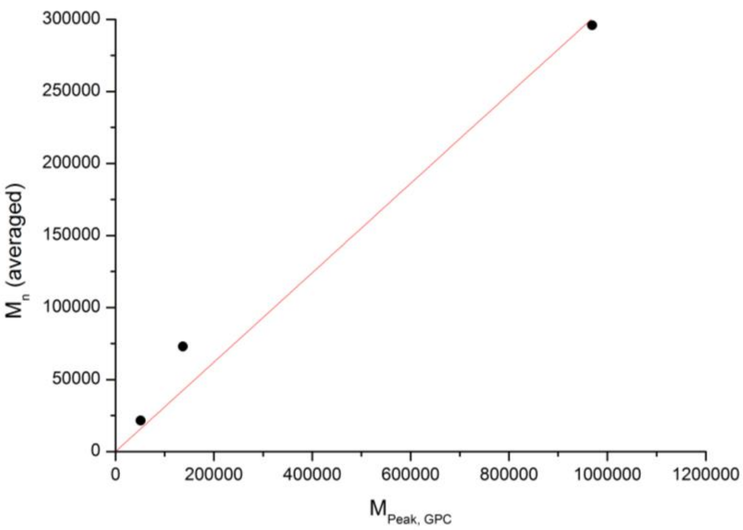

Fig. 3: Linear correlation between overall averaged molar masses M_n and peak molar masses from GPC.

Additional Information: RAFT method

Experimental Procedures

Synthesis of poly(acrylic acid-co-choline acrylate) using dithiobenzoic acid-1-(6,8-disulphonaphthalen-2-ylcarbamoyl)-ethylester disodium salt as RAFT reagent

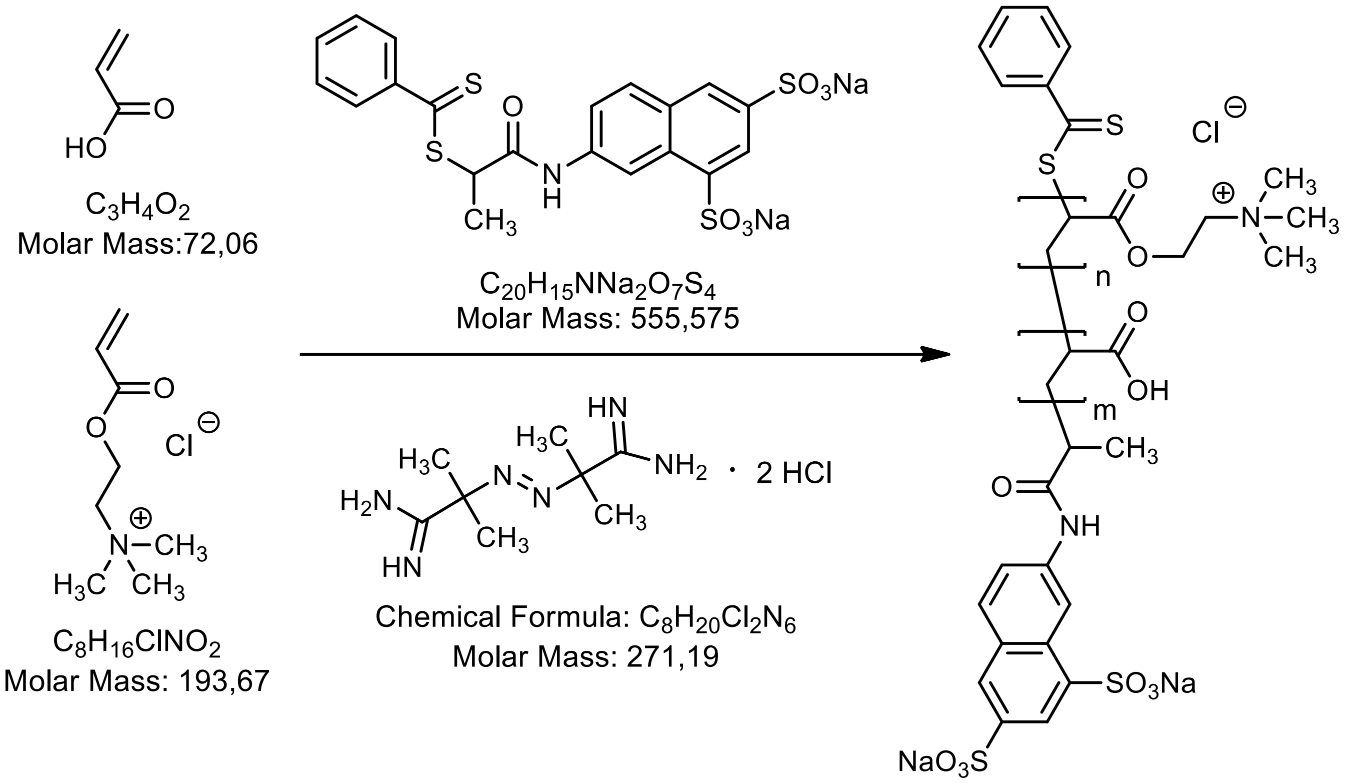

$n_{monomer, total}$	sample code	M_{target}	n_{RAFT} [mmol]	m_{RAFT} (61 wt %)*[mg]	n_{ini} [mmol]	m_{ini}	remark
8.00 g of acrylic acid (111 mmol), 0.28 g of choline acrylate (1,2 mmol)	SaSt140317CS	20000	0.71	650	0.53	143 mg	
	SaSt140317BS	75000	0.19	173	0.14	38 mg	
	SaSt140317AS	200000	0.07	66	0.05	15 mg	7 mg of initiator were added after two days, because only little reaction occurred to this point (^{1}H-NMR)

* 61 wt% of active substance

Additional Information: RAFT method

Details of the Synthesis:
8.0 g of freshly distilled acrylic acid (0.111 mol) and 0.28 g of choline acrylate (1.2 mmol) were dissolved in 26 ml of demineralised water in a 50 ml round flask sealed with a septum. Depending on the target molar mass, adequate amounts of RAFT reagent and initiator V-50 (Wako) were added (see table above). The reaction mixture was degassed by bubbling nitrogen for 20 minutes. While stirring with a magnetic stirrer, the reaction mixture was heated to 65°C in an oil bath for 36 h (for SaSt140317AS: 48 h + 24 h after addition of initiator). The products were purified by dialysis (Roth CelluTrans, cut off 4000-6000) and then isolated by freeze-drying.

Yields:

SaSt140317AS	6.3 g (76%)
SaSt140317BS	6.7 g (81%)
SaSt140317CS	7.5 g (91%)

Analytics: ^{1}H-NMR in D_2O, UV/VIS in water, GPC (details see below)

The presence of choline acrylate moieties in the polymer is evidenced by the signals at 3.2 and 3.8 ppm. Another signal is present at 4.6 ppm, but it is hardly visible, because of superposition with the strong HDO signal at 4.8 ppm. Comparison of the integrals of the signals at 3.2 and 3.8 ppm to the integrals of the polymer main chain (2.7 to 1.3 ppm) confirms a molar ratio of acrylic acid to choline acrylate units in the polymers of approx. 100:1, as desired.

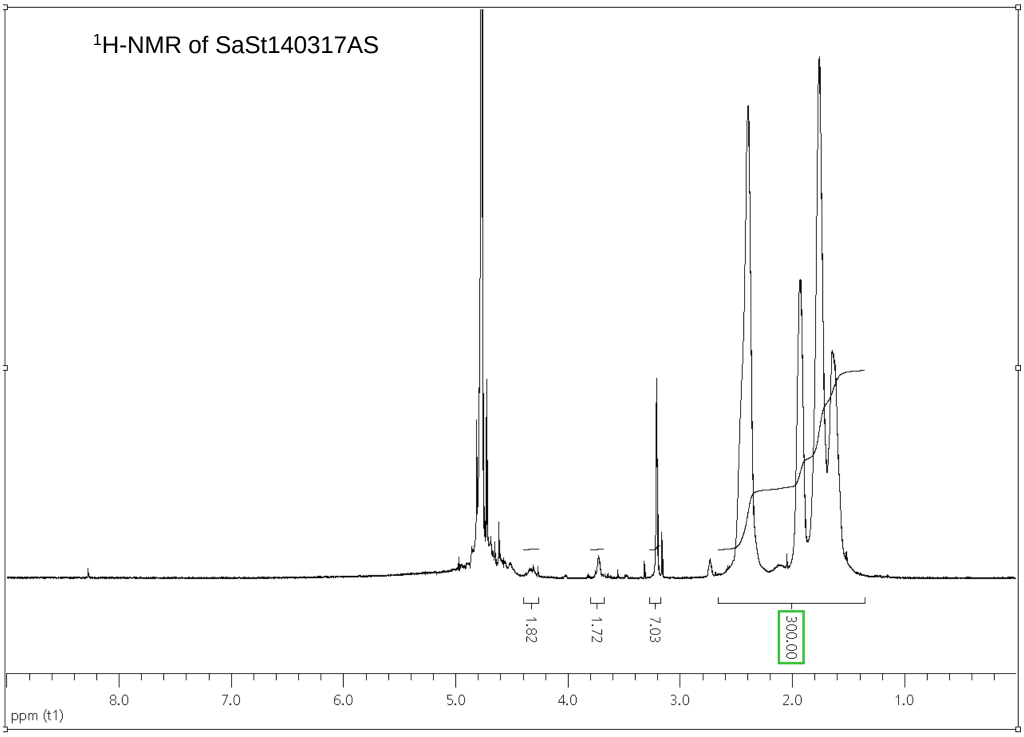

Additional Information: RAFT method

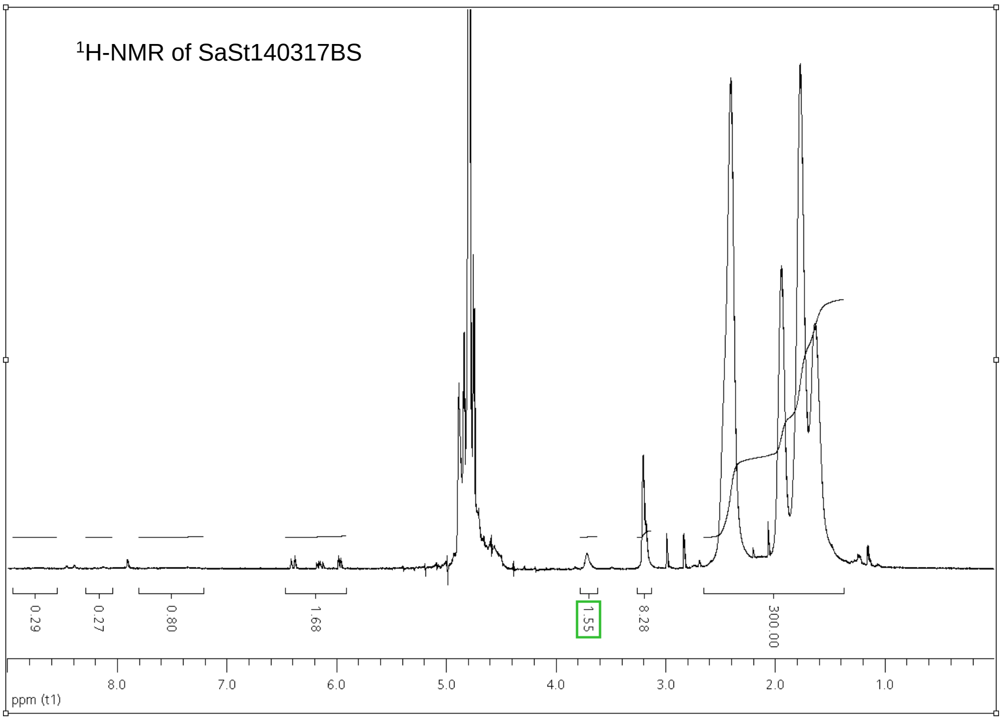

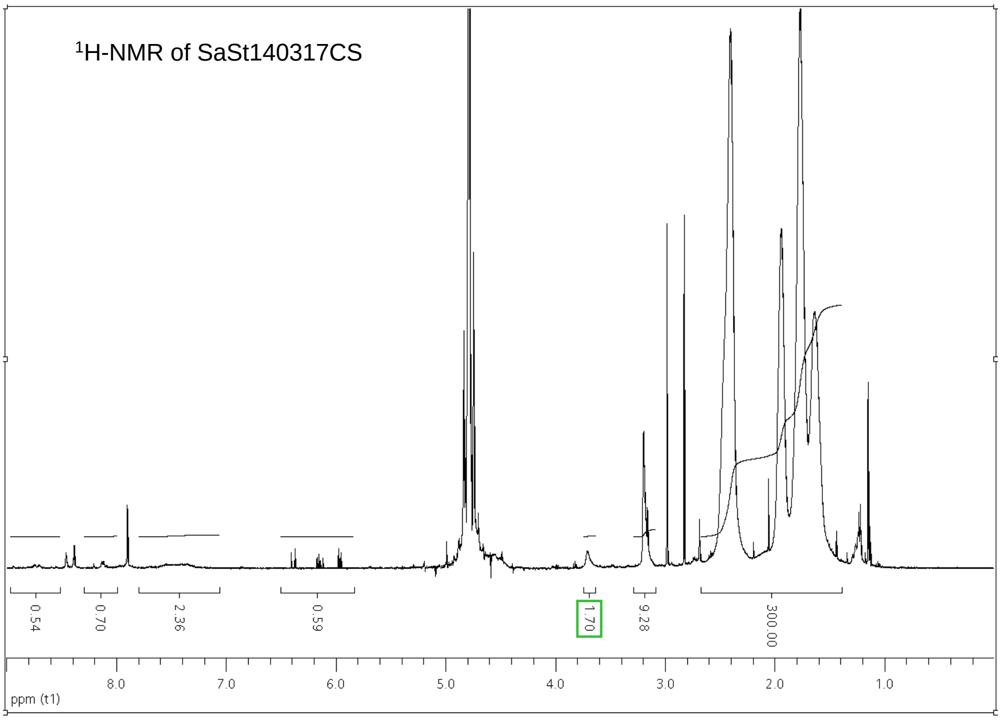

Additional Information: RAFT method

Instrumental parameters of GPC for the characterisation of calibration polymers

- Online Degasser WGE SEC-3010
- Pump WGE SEC-3010
- Eluent: 0,07M Na_2HPO_4 + 10 ppm Na-azide, 0,2 µm filtr.
- Flow: 1 ml/min
- Autosampler WGE SEC-3010, sample volume 111 µl
- Column oven: WGE SEC-3010, 30°C
- Columns: PSS Suprema Guard (50 x 8 mm) + 3000 + 1000 + 100 (300 x 8 mm, 20 µm)
- Calibration standards : Agilent Polyacrylic Acid-Na Salt 1.25 kD – 1100 kD
- Detector: Dual detector WGE SEC-3010 (RI and UV)
- Evaluaton software ParSEC (Brookhaven Instruments)
