# Supplementary material for: Cationic polyacrylamide copolymers (PAMs): environmental half life determination in sludge-treated soil
Source: Environ Sci Eur. 2018 May 18;30(1):16. doi: 10.1186/s12302-018-0143-3 (PMC5959997; doi:10.1186/s12302-018-0143-3)
Supplement: Supplementary file 2 — Additional file 2: Table S1. Soil characteristics of RefeSol 01-A. Table S2. Amount of leachate collected in the course of the lysimeter experiment. Table S3. Measured and calculated data for radioactivity content in the upper soil layers. Table S4. Input data (% aR) for calculation of kinetic parameters by means of CAKE. Table S5. Ultimate degradation in the top 10 cm layer calculated based on experimental data. [file 12302_2018_143_MOESM2_ESM.docx]

Additional file 2: Table S1: Soil characteristics of RefeSol 01-A

| soil | Sand  2000 - 63 µm  (%) | Silt  63  - 2 µm  (%) | Clay  < 2 µm  (%) | Sand  2000 - 50 µm  (%) | Silt  50 - 2 µm  (%) | Clay  < 2 µm  (%) | Corg  (%) | pH  (0.01 M CaCl_2_) | CEC_eff_  (mmol_c_ /kg) | WHC_max_  (g/kg) |
| --- | --- | --- | --- | --- | --- | --- | --- | --- | --- | --- |
|  | according to E DIN ISO 11277 | | | according to USDA | | |  |  |  |  |
| RefeSol 01-A (silty loam) | 76.7 | 17.2 | 6.1 | 76.6 | 17.7 | 5.7 | 0.8 | 5.33 | 17.9 | 291 |

Additional file 2: Table S2: Amount of leachate collected in the course of the lysimeter experiment

| Collection date | Volume [L] |
| --- | --- |
| 27.11.2012 | 60 |
| 19.12.2012 | 90 |
| 26.12.2012 | 85 |
| 31.01.2013 | 90 |
| 07.03.2013 | 82 |
| 19.04.2013 | 85 |
| 03.06.2013 | 85 |
| 19.08.2013 | 24 |
| 29.10.2013 | 93 |
| 18.12.2013 | 120 |
| 07.01.2014 | 120 |
| 30.01.2014 | 85 |
| 20.02.2014 | 72 |
| 13.05.2014 | 82 |
| 02.06.2014 | 83 |
| 07.07.2014 | 60 |
| 15.07.2014 | 65 |
| 31.07.2014 | 35 |
| 28.08.2014 | 60 |
| 09.10.2014 | 73 |
| 27.10.2014 | 60 |
| 17.12.2014 | 110 |
| 30.12.2014 | 87 |
| 16.01.2015 | 115 |
| 17.02.2015 | 82 |
| 04.03.2015 | 68 |
| 09.04.2015 | 85 |
| 06.08.2015 | 70 |
| 08.10.2015 | 90 |
| 07.12.2015 | 150 |

Additional file 2: Table S3: Measured and calculated data for radioactivity content in the upper soil layers.

| Soil layer [cm] | Day after treatment [DAT] | | | | | | |
| --- | --- | --- | --- | --- | --- | --- | --- |
|  | 1 | 183 | 365 | 578 | 730 | 913 | 1095 |
| [Bq / g soil dw] | | | | | | | |
| 0 - 5 | 3315 ± 15 | 3580 ± 60 | 3074 ± 88 | 2822 ± 16 | 2244 ± 20 | 2500 ± 206 | 2143 |
| 5 - 10 |  | 807 ± 53 | 166 ± 8 | 645 ± 36 | 445 ± 12 | 903 ± 11 |  |
| 0 - 10 | 1657 | 2194 | 1620 | 1733 | 1345 | 1701 | 1072 ± 61 |
| [% aR] | | | | | | | |
| 0 - 10 | 100 | 132.4 | 97.8 | 104.6 | 81.2 | 102.7 | 64.7 |

The calculated data are highlighted grey.

Additional file 2: Table S4: Input data [% aR] for calculation of kinetic parameters by means of CAKE

| replicate | Day after treatment [DAT] | | | | | | |
| --- | --- | --- | --- | --- | --- | --- | --- |
|  | 1 | 183 | 365 | 578 | 730 | 913 | 1095 |
| [% aR] | | | | | | | |
| 1 | 100.0 | 131.6 | 95.2 | 106.2 | 82.2 | 110.2 | 64.7 |
| 2 | 100.0 | 134.5 | 97.5 | 104.5 | 80.5 | 99.3 | 64.7 |
| 3 | 100.0 | 131.1 | 100.5 | 103.1 | 80.8 | 98.6 | 64.7 |

Additional file 2: Table S5: Ultimate degradation in the top 10 cm layer calculated based on experimental data.

| DAT [d] | DAT [a] | remaining [% aR] | degraded [% aR] |
| --- | --- | --- | --- |
| 0 | 0 | 100.0 | 0.0 |
| 365 | 1 | 88.0 | 12.0 |
| 730 | 2 | 77.5 | 22.5 |
| 1095 | 3 | 68.2 | 31.8 |
| 1460 | 4 | 60.0 | 40.0 |
| 1825 | 5 | 52.8 | 47.2 |
| 1980 | 5.4 | 50.0 | 50.0 |
| 2190 | 6 | 46.5 | 53.5 |
| 2555 | 7 | 40.9 | 59.1 |
| 2920 | 8 | 36.0 | 64.0 |
| 3285 | 9 | 31.7 | 68.3 |
| 3650 | 10 | 27.9 | 72.1 |
